# Influence of Botulinum Toxin Therapy on Postural Control and Lower Limb Intersegmental Coordination in Children with Spastic Cerebral Palsy

**DOI:** 10.3390/toxins5010093

**Published:** 2013-01-11

**Authors:** Marc Degelaen, Ludo de Borre, Eric Kerckhofs, Linda de Meirleir, Ronald Buyl, Guy Cheron, Bernard Dan

**Affiliations:** 1 Centre d’Analyse du Mouvement, Hôpital Brugmann, Université Libre de Bruxelles (ULB), Brussels B-1020, Belgium; E-Mails: marc.degelaen@huderf.be (M.D.); ludo.deborre@huderf.be (L.B.); 2 Revalidatie Ziekenhuis Inkendaal, Vlezenbeek B-1602, Belgium; 3 Hôpital Universitaire des Enfants Reine Fabiola, Université Libre de Bruxelles (ULB), Brussels B-1020, Belgium; 4 Department of Rehabilitation Research, Vrije Universiteit Brussel (VUB). Brussels B-1020, Belgium; E-Mail: ekerckhofs@vub.ac.be; 5 Universitair Ziekenhuis Vrije Universiteit Brussel (VUB), Brussels B-1020, Belgium; E-Mail: linda.demeirleir@uzbrussel.be; 6 Dienst Biostatistiek en Informatica, Faculteit Geneeskunde en Farmacie, Vrije Universiteit Brussel (VUB), Brussels B-1020, Belgium; E-Mail: rbuyl@vub.ac.be; 7 Unité de Recherche de Neurophysiologie et de Biomécanique du. Mouvement (CP 168), Faculté des Sciences de la Motricité, Université Libre de Bruxelles (ULB), Brussels B-1070, Belgium; E-Mail: gcheron@ulb.ac.be

**Keywords:** Botulinum toxin, cerebral palsy, postural control, intersegmental coordination

## Abstract

Botulinum toxin injections may significantly improve lower limb kinematics in gait of children with spastic forms of cerebral palsy. Here we aimed to analyze the effect of lower limb botulinum toxin injections on trunk postural control and lower limb intralimb (intersegmental) coordination in children with spastic diplegia or spastic hemiplegia (GMFCS I or II). We recorded tridimensional trunk kinematics and thigh, shank and foot elevation angles in fourteen 3–12 year-old children with spastic diplegia and 14 with spastic hemiplegia while walking either barefoot or with ankle-foot orthoses (AFO) before and after botulinum toxin infiltration according to a management protocol. We found significantly greater trunk excursions in the transverse plane (barefoot condition) and in the frontal plane (AFO condition). Intralimb coordination showed significant differences only in the barefoot condition, suggesting that reducing the degrees of freedom may limit the emergence of selective coordination. Minimal relative phase analysis showed differences between the groups (diplegia and hemiplegia) but there were no significant alterations unless the children wore AFO. We conclude that botulinum toxin injection in lower limb spastic muscles leads to changes in motor planning, including through interference with trunk stability, but a combination of therapies (orthoses and physical therapy) is needed in order to learn new motor strategies.

## 1. Introduction

Cerebral palsy (CP) is a developmental central nervous system disorder defined as a group of persistent postural or movement dysfunctions secondary to a non-progressive lesion in the developing brain; the motor disorders of CP are often accompanied by disturbances of sensation, perception, cognition, communication, behavior, by epilepsy and by secondary musculoskeletal problems [1]. CP can be classified according to the motor components. Spastic forms are the commonest; indeed, CP is the most common cause of spastic movement disorders in children [2]. They are characterized by imbalances in muscle activity across joints [3]. With growth, the imbalance between agonists and antagonists often progresses to muscle contracture, joint and bony deformities [1,3,4]. Management in the period before the development of fixed contractures should effectively prevent fixed deformities while being cost-effective and should not result in any serious complications [5]. In the context of comprehensive management of children with spastic forms of CP, botulinum toxin type A (BTX) has been used as a means to decrease spasticity and thereby facilitate functional therapy objectives and orthotic bracing [6,7]. Studies demonstrated that following BTX injection, children with spastic CP can learn some specific targeted movement pattern advised by their physiotherapists [8]. Thus, BTX appears to be a useful option in the interdisciplinary care of children with CP, particularly at a young age so that contractures can be prevented and the motor potential can be optimized [9,10].

The European consensus updated in 2009 summarizes the current understanding regarding an integrated, multidisciplinary treatment approach using BTX for the treatment of children with CP [10]. Treatment planning is centered on improving function, comfort and care, reducing pain, and preventing or correcting deformity. 

In adult acquired conditions, such as stroke, BTX injections into muscles showing spasticity have been shown to improve intralimb coordination of the paretic limb [8]. Here, we aimed to study the effect of BTX on postural control and lower limb intersegmental coordination in children with spastic CP. To this effect, we studied groups of children with CP characterized as either spastic diplegia or spastic hemiplegia, with autonomous walking skills. We recorded tridimensional trunk kinematics, thigh, shank and foot elevation angles, and interjoint coordination in a number of walking paradigms. Namely, the children walked barefoot and with ankle-foot orthoses (AFO), and recordings were performed prior to BTX administered according to a management protocol (see Section 4.2) as well as four months after the injections. We found that BTX induced changes in lower limb intersegmental coordination and resulted in increased trunk movements in the transverse plane. Wearing AFO further altered hip-knee coordination during the swing phase of gait and increased trunk movements in the frontal plane. This suggests that the lower limb motor control strategies that emerge after BTX, enhance the trunk postural challenge. This needs to be taken into account when planning rehabilitation management.

## 2. Results

Mean age, height and body weight were 7.6 ± 3.0 years, 124.64 ± 17.1 cm, 25.57 ± 6.4 kg and 5.6 ± 3.2 years, 115.07 ± 16.8 cm, 23.00 ± 8.4 kg for children with diplegia and hemiplegia, respectively. All children completed the locomotor task.

### 2.1. Trunk Movements

Descriptive data of trunk motion with respect to the extracorporeal space (laboratory reference, see Section 4.4) are presented in Table 1 and Table 2. In the barefoot condition, children with spastic diplegia showed an increased range of trunk motion in the frontal plane and a decrease in the sagittal plane, whereas children with spastic hemiplegia showed a decrease in trunk motion in both these planes after BTX. In the transverse plane, both groups showed significant increase trunk motion. In the AFO condition, trunk motion increased significantly in the frontal plane in both groups but no significant changes were found in the other planes.

**Table 1:** Kinematic features in the barefoot condition.

	Spastic diplegia		Spastic hemiplegia	
*n* = 24	Pre-BTX	Post-BTX	Pre-BTX	Post-BTX
Trunk sag. (ROM, deg)	8.9 ± 4.4	8.5 ± 4.0	10.9 ± 13.2	6.2 ± 2.3
Trunk front. (ROM, deg)	5.6 ± 2.7	6.3 ± 3.0	6.2 ± 3.5	5.9 ± 2.2
Trunk trans. (ROM, deg)	12.2 ± 4.7	15.2 ± 7.7	9.1 ± 5.6	12.4 ± 4.6
Variance of PV1 (%)	76.6 ± 4.3	72.9 ± 5.3	77.8 ± 6.0	76.9 ± 4.4
Variance of PV2 (%)	23.1 ± 4.3	26.8 ± 5.3	21.6 ± 5.9	22.7 ± 4.3
Variance of PV3 (%)	0.3 ± 0.2	0.3 ± 0.2	0.6 ± 0.4	0.5 ± 0.2
Covariation plane angle (deg)	10.3 ± 4.7	8.4 ± 4.1	9.6 ± 5.0	7.1 ± 4.2
Minimal relative phase	−124.6 ± 13.4	−124.7 ± 19.7	−143.2 ± 14.3	−140.8 ± 8.1

Mean and standard deviation; * significant difference (*p* < 0.05) between pre- and post-BTX; ** significant difference (*p* < 0.05) between diplegia and hemiplegia.

**Table 2:** Kinematic features in the ankle-foot orthosis condition.

	Spastic diplegia		Spastic hemiplegia	
*n* = 24	Pre-BTX	Post-BTX	Pre-BTX	Post-BTX
ROM Trunk sag (deg)	12.2 ± 5.6	12.3 ± 5.8	9.6 ± 4.7	8.1 ± 3.0
ROM Trunk front (deg)	7.4 ± 1.9	10.0 ± 3.4	5.7 ± 2.0	8.3 ± 2.7
ROM Trunk trans (deg)	22.3 ± 6.0	21.4 ± 6.9	16.3 ± 4.9	17.9 ± 9.7
Variance of PV1 (%)	79.5 ± 6.2	77.3 ± 6.0	77.6 ± 5.9	78.2 ± 3.1
Variance of PV2 (%)	20.4 ± 6.3	22.4 ± 6.3	21.9 ± 6.0	21.5 ± 3.1
Variance of PV3 (%)	0.1 ± 0.2	0.2 ± 0.4	0.4 ± 0.3	0.3 ± 0.2
Covariation plane angle (deg)	10.2 ± 3.0	10.1 ± 4.1	7.8 ± 4.7	9.7 ± 5.2
Minimal Relative Phase	−131.2 ± 12.0	−140.9 ± 10.9	−143.6 ± 9.1	−146.6 ± 7.5

Means and standard deviation; * significant difference (*p* < 0.05) between Pre and Post Botox; ** significant difference (*p* < 0.05) between diplegia and hemiplegia.

### 2.2. Lower Limb Coordination

The specific coordination pattern of lower limb intersegmental coordination (evaluated with planar covariation of elevation angles, see Section 4.4) showed significant changes after BTX in the barefoot condition but not in the AFO condition (Table 1 and Table 2). In the barefoot condition, planarity of the covariation of the elevation angles of the thigh, shank and foot over the gait cycle (quantified by the percentage of variance accounted by the third eigenvector, PV3) remained generally unaltered in both groups: 0.3% ± 0.2% for the children with spastic diplegia and 0.56% ± 0.4% for those with spastic hemiplegia, suggesting that the complexity in intersegmental lower limb coordination was not significantly changed following BTX. The covariation plane angle decreased in both groups. However, there was reorganization of this coordination within the preserved covariation planarity. PV1 decreased significantly after BTX while PV2 increased.

**Figure 1:**
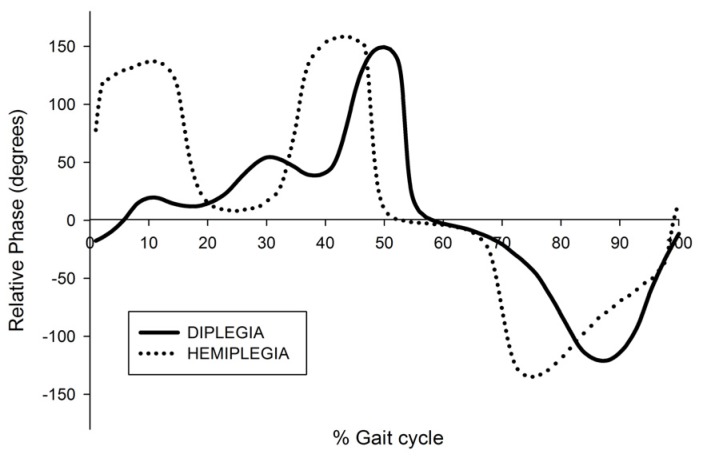
Relative phase plots in the barefoot condition before botulinum toxin infiltration. For one child with diplegia and one with hemiplegia.

**Figure 2:**
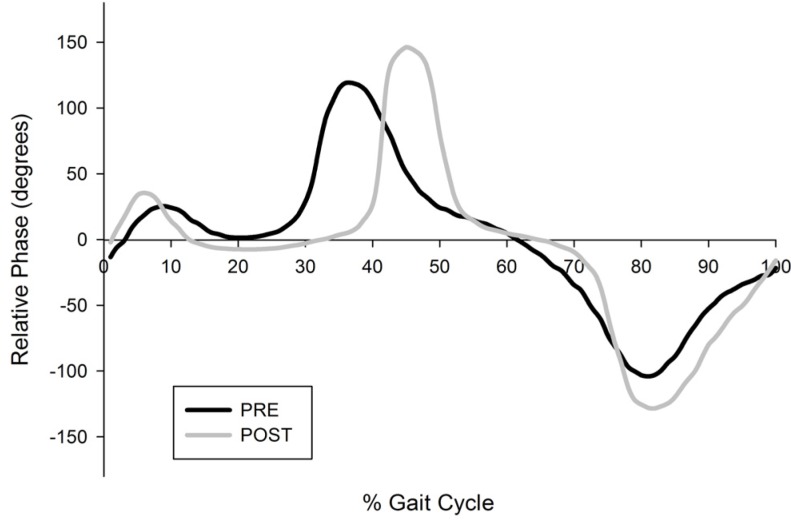
Relative phase plots in the ankle-foot orthoses condition before and after botulinum toxin. For one child with diplegia.

As regards interjoint coordination of the hip and knee during the swing phase, the minimal relative phase in swing was significantly lower in the diplegia group (−124.64 ± 13.4) compared to the hemiplegia group (−143.23 ± 14.3) in the barefoot condition, pre- and post-BTX (Figure 1 and Figure 2). However, BTX did not change the minimal relative phase within each group. In the AFO condition, there was a significant increase of the minimal relative phase in both the diplegia and hemiplegia groups.

## 3. Discussion

### 3.1. Trunk Stability

The development of trunk stability is essential to the control of walking [11]. In typical development, direction-specific adjustments emerge early in infancy [12,13], followed by fine tuning afforded by integration of multimodal sensory input [14]. In children with CP, postural control is impaired from an early age [14,15,16]. AFO are commonly prescribed in order to improve spatio-temporal gait parameters, alignment of the knee, control the development of equinus deformity and to reduce energy expenditure [17,18,19]. However, they have been shown to induce or enhance trunk movements, which are interpreted as compensatory motion [20,21]. In addition to increased trunk excursions, increased pelvic excursions are commonly found during gait in children with CP compared to typically developing children, as described in the present study [20]. This contributes to postural instability [17]. In our study, the use of AFO was associated with increased stability in the distal joints, which was related to improved pelvic stability, but it was also associated with increased trunk motion. The latter might be considered as a compensatory strategy for the reduced ankle power generation at push-off, caused by AFO, combined with increased step length and walking velocity [20]. Furthermore, AFO improved intralimb coordination during gait in children with spastic diplegia. However, wearing AFO results in increased trunk motion, which may be problematic in the context of difficult postural control [21]. Even in typically developing children, wearing solid AFO induced compensations at the pelvis and trunk in the frontal, sagittal and transverse planes in order to assist in limb progression and clearance [22]. Although this study was performed with AFO, the authors suggested that many of these compensations should be expected to occur in children with restricted ankle motion resulting from other causes than AFO use [22].

The effect of BTX on trunk stability has not been the focus of much study. Here, we found that BTX injection in the lower limbs does influence trunk control during gait in children with spastic CP. The effect manifested mostly in the transverse plane when the children walked barefoot and in the frontal plane when they walked with AFO. Gait characteristics of these children are habitually determined largely by spasticity in muscles whose action is marked in the sagittal (psoas, hamstring, gastrocnemius) and frontal (hip adductors) planes. By reducing muscle tone in these muscles, BTX results in use of alternative strategies with increased degree of freedom with trunk rotation in the transverse plane. However, when AFO are worn, mechanical constraint prevents forward tibial progression in stance so that foot clearance may be realized through a strategy that involves a contralateral trunk inclination in the frontal plane.

### 3.2. Lower Limb Kinematics

The planar coordination rule was previously demonstrated in healthy adults as well as in a number of conditions with gait impairment, including Parkinson’s disease [23], spastic paraparesis [24], stroke [8] and transfemoral prosthetic gait [25]. This coordination rule is, however, not obligatory, and it emerges developmentally [26]. Here, we show that it is present in both spastic diplegia and hemiplegia, whether with or without AFO. BTX injection in the lower limbs was associated with a change in the covariation plane angle. This might reflect changes in mechanical energy cost [27], which was otherwise demonstrated in BTX therapy of children with spastic forms of cerebral palsy [28,29]. The reorganization of the covariation of the elevation angles of lower limb segments in the sagittal plane following BTX likely reflects adaptive strategies with preservation of the planar covariation rule. 

It could be hypothesized that the differences we found in minimal relative phase between children, with diplegia and those with hemiplegia are consistent with better overall selectivity in lower limb in unilateral cerebral palsy [30]. Although we did not specifically evaluate lower limb selective motor control in this study, another study did not find any group differences in this regard between hemiplegic and diplegic cerebral palsy [31]. 

Our finding of alteration of the specific intralimb coordination pattern in the barefoot condition but not in the AFO condition, raises the question of the constraining effect of orthoses on the emergence of adaptive motor strategies. Such an effect was previously described in young children with typical development (4–5 years of age) and contrasted with significant changes in intralimb coordination in older children with typical development (9–10 years) [32]. This suggests that in children with cerebral palsy, as in young children with typical development, reducing the degrees of freedom in the lower limbs may limit the emergence of selective coordination. This calls for caution when this approach is considered.

## 4. Experimental Section

### 4.1. Study Population

#### Patients

Twenty-eight ambulatory children aged between 3 and 12 years (7.5 ± 1.9) with spastic CP recruited from our Reference Center for Cerebral Palsy (CIRICU). Inclusion criteria were as follows: diagnosis of spastic CP ascertained within the Reference Center, age between 3 and 12 years, GMFCS level I or II, no history of orthopedic surgery and no botulinum toxin injections within the last 12 months. Fourteen children (3 girls, 11 boys) had a bilateral form (spastic diplegia) and 14 (4 girls, 10 boys) had a unilateral form (spastic hemiplegia). Clinical features are summarized in Table 3. All children received 1–5 sessions of physical therapy per week at baseline and 3–5 weekly sessions for 6 weeks following BTX injection. 

**Table 3:** Clinical features of participants.

Pt	Age (year)	Gender	CP type	Gestage (week)	Indep. walking	GFMCS	Cognitive impairment	Epilepsy	Injected muscles	BTX dose (U)
1	4	M	R Hemi	39	16 m	II	None	No	ham, gastr	11
2	3	F	R Hemi	42	14 m	I	mild	No	gastr, sol	8
3	4	F	R Hemi	38	21 m	II	None	No	ham, gastr	16.5
4	5	M	R Hemi	38	14 m	II	None	No	ham, gastr	12
5	12	M	R Hemi	39	24 m	I	None	No	ps, add, ham, gastr	15
6	3	M	R Hemi	40	?	I	None	No	ham, gastr, sol	12
7	4	M	R Hemi	33	21 m	I	Mild	C	add, ham, gastr	12.5
8	11	M	R Hemi	40	?	II	None	No	ham, gastr	12
9	10	M	R Hemi	40	18 m	I	None	No	ham, gastr	11
10	4	F	R Hemi	40	?	I	None	No	gastr, sol	6.5
11	3	M	R Hemi	?	?	I	None	No	ham, gastr, sol	12
12	5	F	R Hemi	30	18 m	I	None	No	ham, gastr	14
13	6	M	L Hemi	28	?	I	None	No	ham, gastr, sol	15
14	4	M	L Hemi	40	12 m	II	None	No	add, ham, gastr, sol	14
15	4	F	Di	39	15 m	I	Mild	No	ps, add, ham, gastr	24
16	5	M	Di	35	16 m	II	None	No	ps, ham, gastr	18
17	12	F	Di	33	24 m	II	Severe	No	ps, ham	15
18	6	M	Di	35	11 m	II	None	No	ps, add, ham, gastr	29
19	12	M	Di	26	24 m	II	Mild	No	ham, gastr	18
20	8	M	Di	25	24 m	II	None	No	ps, add, ham, gastr	15
21	3	F	Di	37	24 m	II	None	No	add, ham, gastr	28
22	8	M	Di	41	27 m	I	None	No	ps, add, ham, gastr	16
23	6	M	Di	40	20m	II	None	No	ham, gastr	20
24	7	M	Di	40	?	II	None	No	add, ham, gastr	17.5
25	9	M	Di	28	?	I	None	No	ps, add, ham, gastr	21
26	6	M	Di	40	48 m	II	None	No	ham, gastr	18
27	8	M	Di	40	30 m	II	None	No	add, ham	16.5
28	10	M	Di	?	?	I	None	No	ham, gastr	16

M = male, F = female, Di = diplegia, Hemi = hemiplegia, R = right, L = left, ps = psoas; ham = hamstrings, add = adductors, gastr = gastrocnemius, sol = soleus, C = controlled with medication, U = Units.

### 4.2. Botulinum Toxin Injection

BTX (Botox, Allergan, Irvine, CA, USA) was injected intramuscularly by sterile technique using a 23-gauge needle into muscles of the lower limbs showing spasticity (psoas, hip adductors, medial hamstrings, gastrocnemius, soleus) in all children in both groups (those with spastic diplegia and those with spastic hemiplegia). The choice of muscles selected for injection was determined following multidisciplinary discussion taking into account the degree of spasticity on clinical testing, the presumed effect of spasticity on function as evaluated by instrumented gait analysis (including kinematics, kinetics and muscle activation patterns) and the expected short-term goal of the physiotherapist. Correct needle placement in the area of the neuromuscular junction [33] was tested by passive mobilization of the relevant joints. BTX was injected after negative pressure was applied to ensure that the needle tip was extravascular. The dose of BTX given to each child in individual muscles is shown in Table 3. The doses of BTX we used ranged between 8 and 29 Units These doses are in keeping with those recommended in the updated European Consensus 2009 on the use of Botulinum toxin for children with cerebral palsy [10]. Children with hemiplegia were infiltrated in both gastrocnemius and soleus muscles (mean dosage was 6 Units/kg and 2 Units/kg for gastrocnemius and soleus, respectively. Children with diplegia were infiltrated only in gastrocnemius muscles with a mean dosage of 6 Units/kg.

### 4.3. Locomotor Task and Recording

**Figure 3:**
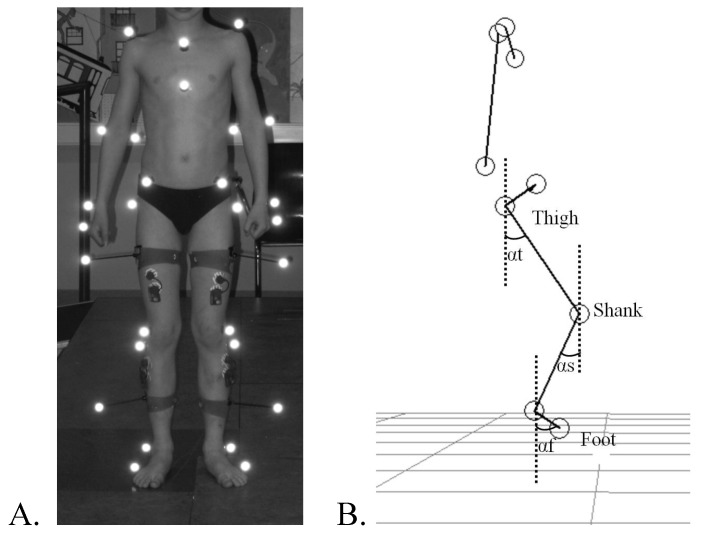
Marker placement and angle definition for lower limb segments coordination. (**A**) Self-reflective markers positioned on anatomic landmarks for kinematic recording; (**B**) Definition of the elevation angles of the thigh (αt), shank (αs) and foot (αf).

3D gait recording was performed before BTX injection and 4 months after the injection. Participants were barefoot and wore tight pants or light cloths that would not cover the markers or interfere with the movement. They were instructed to walk as naturally as possible over an 8-meter walkway at a comfortable speed, looking straight forward. The task was recorded using the optoelectronic ELITE system (BTS, Milan, Italy) following a standard protocol [32]. This system consists of six charge-coupled device cameras detecting retro-reflective markers using a sampling rate of 100 Hz. The 26 markers were placed on the subjects’ skin overlying the following anatomical landmarks: C7, the acromion process, Th2 spinous processes, Th10 spinous processes, xyphoid process, suprasternal notch, sacrum, anterior superior iliac spines, greater trochanters, thighs (midpoint between greater trochanter and lateral head of the femur), knees (lateral head of the femur and fibula), shank (midpoint between the lateral head of the fibula and the lateral malleolus), lateral malleoli, second metatarsal heads, and heels (Figure 3A). 

### 4.4. Kinematic Analysis

**Figure 4:**
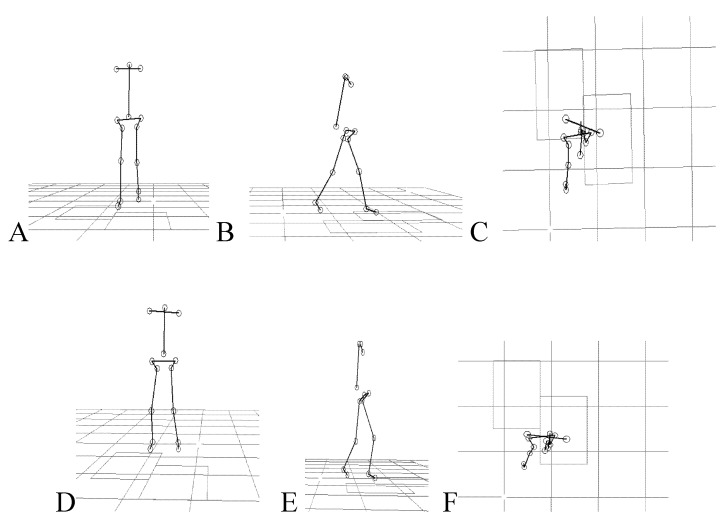
Kinograms (stick diagrams). Shown kinograms represent a 5 year-old child with right hemiplegia walking barefoot in a (**A**) frontal; (**B**) sagittal and (**C**) transverse plane views and a 7 year-old child with diplegia in the (**D**) frontal; (**E**) sagittal and (**F**) transverse planes of locomotion.

Temporal spatial measurements were computed for each trial using the Eliclinic software (BTS, Milan, Italy). Kinematic data of the trunk were smoothed and processed with SMART analyzer (BTS, Milan, Italy) for time normalization and to calculate average positions, distances and angles. Space calibration was oriented such that the X-axis was aligned with the walking direction (positive direction), the Y-axis was vertical and the Z-axis was transversal to the walking direction, the planes used to study kinematic data were defined as follows: sagittal plane (XY), frontal plane (YZ) and horizontal plane (XZ). After reconstruction of the stick diagrams representing successful locomotion of subjects (Figure 1B and Figure 4), we focused our analysis on the orientation of the trunk and the lower limb segments with respect to the vertical. The following segments were analyzed: trunk (defined by the line connecting the acromion and the iliac spine markers), thigh (trochanter-knee), shank (knee-lateral malleolus), and foot (lateral malleolus-fifth metatarsal). The elevation angles of the thigh, shank, and foot in the sagittal plane are noted αt, αs, and αf, respectively (Figure 3B).

The methods for analyzing the planar covariation of elevation angles were the same as those used in previous work [26,27,28,29,30,31,32,33,34,35,36,37,38,39,40,41]. Briefly, the statistical structure underlying the distribution of the geometrical configurations associated with the observed changes of the elevation angles was described by principal component (PC) analysis. The PCs were computed by pooling together the samples of time-varying angles after subtraction of the mean value, and identified the best-fitting plane of angular covariation for each session. 

Relative phase (RP) analysis was used to quantify interjoint coordination of the hip and knee during the swing phase of gait. Hip-knee angle diagrams were plotted for each limb and analyzed as described elsewhere [30,41], with specific focus on the portion of the swing phase in which simultaneous hip flexion and knee extension normally occur. 

Mean ± SD were used to describe both quantitative characteristics of the test subjects as well as quantitative outcome parameters. Statistical analysis was performed using a repeated measures ANOVA to examine the within group (form) and between group (spastic diplegia *vs.* spastic hemiplegia) differences. The dependent variables were RPs, PCs, angle of covariation plane. A Pearson Correlation Coefficient was used to examine the linear relation between two quantitative variables, namely body weight and body length. The α level was set at 0.05 for all statistical procedures, which were conducted using the Statistical Package for Social Sciences v. 20 (IBM, Armonk, NY, USA). 

### 4.5. Ethical Aspects

The study protocol was approved by the institutional ethics committee and informed consent obtained from each subject and legal guardian.

## 5. Conclusions

We conclude that BTX allow children with CP to change motor coordination in the lower limbs and also interferes with trunk stability. The lower limb motor control strategies that emerge after BTX enhance the trunk postural challenge. This needs to be taken into account when planning rehabilitation management. In order to have the best results in BTX treatment, combination therapy with orthoses and physical therapy is needed in order to learn new effective motor strategies.
